# Book review: *Practical Control of Mosquito Disease Vectors*

**DOI:** 10.5281/zenodo.15130501

**Published:** 2025-04-03

**Authors:** Bart G.J. Knols

**Affiliations:** 1 Editor, MalariaWorld Journal

## Abstract

Vector-borne diseases are becoming increasingly difficult to control worldwide due to factors such as insecticide resistance and climate change, which facilitate their spread and alter transmission dynamics. As conventional tools fail more frequently, the demand for innovative vector control strategies continues to grow. In this book, Charlwood and co-authors review current and emerging methods for controlling disease vectors, with a particular focus on malaria mosquitoes. The book serves as an introduction for new-comers to the field, featuring chapters on mosquito ecology and sampling techniques. At the same time, it offers seasoned professionals an opportunity to stay up to date with the latest advancements. *Culture and Vector-Borne Disease* and *The Role of Politics in Vector Control* offer thought-provoking insights and are essential reading for both novice researchers and experienced control practitioners.

Charlwood and co-authors start their book with a rather bleak picture of the world: Wars, climate change and the imminent collapse of biodiversity if we’re not changing our course of action soon. They argue that anything we do in vector control should have safe-guarding of the planet, its climate and biodiversity, in mind. The book builds on the DDT paradigm: **D**elight (we have something new!), **D**espair (Shoot, it’s not working anymore!) And **T**ry something new. The chapters that follow engage with this latter component mostly.

Chapters, on mosquito life-history, a brief history of malaria control, and sampling of mosquito vectors follow. In a fast pace, topics like mosquito speciation, invasive mosquitoes, mating, blood-feeding and sugar-feeding behaviours are described, which will be of interest to the newcomer in the field. The chapter on malaria control lists quinine as the first malaria control tool, which is arguably wrong since the first records of the use of sweet wormwood (*Artemisia annua*) against fevers (likely malaria) in China date back to ~160 BC. Equally, the authors mention that ‘*Before 1944, it was obvious that methods of malaria control by source reduction using larvicides such as kerosine and Paris Green dust were only feasible in urban centres or limited areas’.* By that time, however, the Brazil campaign against *Anopheles gambiae*, based primarily on larviciding using Paris Green, had already been successfully completed (and published [1]). The Gates Foundation was established in 2000, not in 1994. Apart from these minor errors, this short historical overview tells us that many mistakes we made in the past are made time and again, which is quite disturbing. Reaching WHO’s malaria targets by 2030 is rightly considered as impossible. The chapter on sampling provides a nice overview of methods that can be used for indoor or outdoor resting/ feeding mosquitoes, although Charlwood focuses much on his own inventions rather than those most broadly used today.

The chapter on drone technology for surveillance provides a great introduction (with many useful references) with two case study details and a balanced discussion on the pros and cons of using drones for surveillance, control, or mosquito releases. The chapter on *Wolbachia* provides an excellent introduction to the various ways the bacterium can be used and is critical about its prospects for disease control in a balanced manner. The chapter on ivermectin, an endectocide, is very well written and highly informative.

It is true that proper house modification probably yields the largest health benefits, and a suite of options is presented, like eave netting, window screening, netting verandah’s, screened doors, etc. Charlwood appears not overly impressed by eave tubes, even though this is likely going to be the only intervention listed by him that may ever be implemented in a programmatic manner. Some of the options presented, like polystyrene beads that are used in pit latrines, are now decades old, and though effective, never got implemented widely. Charlwood offers many interesting options but does not explain why the adoption of these methods by communities is not forthcoming.

Although bednets have contributed significantly to malaria reductions between 2000-2015, problems have arisen since, mostly related to insecticide resistance, lack of financing, and changes in vector behaviour. The chapter on the future of bednets provides a critical view of the current nets, manufacturing outside Africa, lack of domestic markets, and problems with finding suitable actives to address mounting issues with insecticide resistance.

The chapter on attractive toxic sugar baits (ATSBs) summarises findings over the last 15 years and gives some options for future studies. Given that recent results with ATSBs in Africa have not given consistently good results, it remains to be seen where this approach is heading.

Whether or not there is a place for topical repellents in malaria control remains to be seen. Although controlled trials have shown this intervention to be cost-effective, use and compliance remain insurmountable hurdles in many places, especially when the apparent biting density is low but transmission may be intense. From burning plant materials through mosquito coils to the current passive emanator spatial repellents, the latter seem to hold promise and recent evaluations show favourable epidemiological outcomes. Charlwood’s idea to use spatial emanations inside cars and trucks to reduce the spread of invasive mosquitoes is novel and creative and deserves attention.

Old methods, notably the use of larvivorous fish, with surprising good results in some cases, but flawed by pertinent issues that are hard to resolve, like the use of non-native fish species, are described. At best, it seems, fish may play some supplementary role under special circumstances, but overall its role will remain limited. The same applies to zooprophylaxis, i.e., the use of animals as alternative baits (and dead-end hosts), possibly treated with an insecticide or endectocide for which the authors do provide a nice overview of historic field and modelling studies. They conclude, however, that the true merits of this approach remain questionable.

The final two chapters on ‘culture’ and ‘politics’ in vector-borne disease control, authored by Charlwood himself, provide an interesting mix of philosophical prose mixed with the author’s real-life experiences. He points out the shortcomings of ‘community involvement’ in cases where the community remains unclear on the benefits of interventions or have not received adequate information about what interventions will *not* deliver. Laced with his own experiences from Tanzania, Mozambique, São Tomé & Principe and Brazil, Charlwood sums a list of often at best temporary successes but mostly failures and provides, by and large, good explanations about the why. I am not sure though if, as he suggests, impoverished communities in Africa, can be turned into *citizen scientists* in the same way as many Europeans have. It is hardly conceivable that those that find themselves at the bottom of the Maslow pyramid can be enthused to start collecting data on mosquitoes and contribute to the greater good if the pressing needs of the day (food, shelter, etc.) are not met first. In the realm of politics, he pleas for citizens participating in the governance of science. He then ventures into capitalism, blockchain, and the required new economic world order needed to save the planet and uses examples from Venezuela and São Tomé & Principe to demonstrate how strong the impact of political change can be on malaria. I’ll leave it to the readers to build their own opinion regarding the merits of vertically versus horizontally-staged intervention campaigns. From politics surrounding DDT use in The Gambia to social media and the current struggles with misinformation and disinformation, Charlwood wanders off in many directions and lacks a coherent storyline here and there.

Although not as powerful as Desowitz' classic *The Malaria Capers* [1], this book is certainly worth reading and delivers a good and here and there surprising outlook on vector-borne disease control.
